# Unpacking Theory of Mind in developmental coordination disorder: the role of manual dexterity and perspective-taking

**DOI:** 10.3389/fpsyg.2026.1823954

**Published:** 2026-06-05

**Authors:** F. Manzi, G. Purpura, G. Peretti, V. Di Giusto, V. Caravella, D. Massaro, A. Marchetti, A. Cavallini

**Affiliations:** 1 Research Center on Theory of Mind and Social Competence in the Lifespan, Department of Psychology, Università Cattolica del Sacro Cuore, Milan, Italy; 2 IRCCS Fondazione Don Carlo Gnocchi, Milan, Italy; 3 Graduate School of Engineering Science, Osaka University, Osaka, Japan; 4 School of Medicine and Surgery, University of Milano Bicocca, Milan, Italy

**Keywords:** developmental coordination disorder, motor coordination, perspective-taking, social cognition, theory of mind

## Abstract

Developmental Coordination Disorder (DCD) has often been associated with challenges in social functioning, but it remains unclear whether these reflect a generalized impairment in Theory of Mind (ToM) or selective vulnerabilities related to motor and cognitive constraints. This study examined multiple ToM components in 20 children with DCD aged 7–11 years. Participants completed standardized measures of cognitive functioning (WISC-IV), motor coordination (MABC-2), and parent-reported social difficulties (SRS-2), along with a ToM battery assessing perspective-taking, second-order false belief, advanced ToM, and affective ToM. Partial correlations controlling for age, gender, and social symptom severity revealed distinct associations across ToM components. Perspective-taking was positively associated with manual dexterity, whereas affective ToM was related to verbal comprehension, and advanced ToM was associated with overall intellectual functioning, verbal comprehension, and working memory. Second-order false belief performance showed no significant associations. A multiple linear regression model indicated that manual dexterity was the only significant predictor of perspective-taking performance, even after controlling for demographic variables and social symptom severity. These findings suggest that ToM abilities in DCD are not globally impaired but differentially supported by motor and cognitive resources. Perspective-taking appears selectively associated with motor coordination, highlighting the importance of considering this relationship in rehabilitation of children with DCD.

## 1 Introduction

Developmental Coordination Disorder (DCD) is a complex neurodevelopmental disorder characterized by significant impairment in motor control and coordination in the absence of neurological, sensory, or intellectual disabilities (American Psychiatric Association, 2013, 2022). Although core symptoms primarily involve the motor domain and its impact on adaptive functioning, researchers have identified a specific neuropsychological profile in this population. This profile is characterized by difficulties in visuospatial abilities (Pinero-Pinto et al., 2022; van Dyck et al., 2022), executive functions (Asonitou et al., 2025; Sartori et al., 2020), and the social domain (De Roubaix et al., 2024; Lingam et al., 2010; Schoemaker et al., 2024).

Regarding social difficulties, Smyth and Anderson (2000) reported isolation and a preference for solitary play in children with DCD. Similarly, Dewey et al. (2002) found that these children exhibited more internalizing and externalizing behaviours, as well as greater social problems, compared to typically developing peers. In line with these findings, current research highlights that limited participation in group activities—often due to motor difficulties—exacerbates social exclusion and feelings of loneliness, further compromising the quality of peer relationships (Mancini et al., 2024; Omer and Leonard, 2021). Consequently, social challenges in DCD have recently received increased empirical attention. As a matter of fact, evidence suggests an overlap in the social and motor presentations of DCD and other neurodevelopmental disorders, such as autism spectrum disorder (ASD; Miller et al., 2024; Sumner et al., 2016). These partial overlaps with ASD have prompted recent work to examine whether Theory of Mind (ToM) and related socio-cognitive processes may contribute to social functioning in DCD (Kilroy et al., 2021; Kilroy et al., 2022).

In this context, Kilroy et al. (2022) found significant difficulties in social responsiveness in children with DCD, although their impairments in the ability to take other people’s perspective—commonly referred to as ToM—were less severe than those observed in children with ASD. ToM is a complex socio-cognitive ability that enables individuals to understand and predict their own and others’ mental states (e.g., emotions, intentions, and beliefs) in everyday interactions (Bianco et al., 2025; Wellman and Cross, 2001; Yu and Wellman, 2024; Valle et al., 2015). Given its central role in social functioning, ToM represents a relevant framework for investigating socio-cognitive processes in DCD. Developmental evidence suggests that some ToM components—such as the non-verbal attribution of intention—may be related to motor skills (Obeid et al., 2022), although the relationship between more explicit metarepresentational aspects of ToM (e.g., false-belief understanding) and motor functioning remains debated (Kenny et al., 2016). Consistently, a growing body of research highlights the role of embodied and visuospatial processes in specific ToM components, especially perspective-taking (Caldwell et al., 2026; Hirai et al., 2020). Perspective-taking was of particular interest because, although the relationship between ToM and Level-1/Level-2 perspective-taking remains debated, these distinctions help capture the visuospatial demands involved in representing another person’s viewpoint (Flavell, 1974, 1977; Flavell et al., 1981; Surtees et al., 2012). This issue is especially relevant in DCD, where visuospatial and fine-motor difficulties are common and spatial perspective-taking may be fragile (Gauthier et al., 2018; Orefice et al., 2024). This interpretation is consistent with neuroimaging evidence showing reduced connectivity between the inferior frontal gyrus and the ToM network in children with ASD compared to those with DCD (Jayashankar et al., 2023). Moreover, in comparison to DCD group, children with ASD showed hyperconnectivity between the inferior frontal gyrus and the left insula—an area involved in emotional and affective-cognitive abilities. Together, these data support the existence of distinct phenotypes of social impairment across these two disorders. However, while social functioning and its neurobiological correlates are extensively studied in ASD, the literature on social impairment in DCD remains limited and inconsistent. As a result, the specific socio-cognitive mechanisms underlying social difficulties in DCD are still not well understood. Despite these indications, it remains unclear which components of ToM may be selectively associated with motor impairment in this population. This represents a significant gap in the literature and limits the development of targeted and personalised interventions.

The present study aimed to analyse ToM abilities in children with DCD by specifically examining the relationship between motor impairment and distinct ToM components. To address these issues, we administered a comprehensive assessment protocol targeting distinct ToM components, including perspective-taking, second-order false belief, advanced ToM, and affective ToM. Based on previous evidence, we hypothesised that ToM would not be globally impaired in children with DCD but would instead show a differentiated profile across components (Kilroy et al., 2022). Specifically, we expected that components relying more strongly on visuospatial and embodied processes—such as perspective-taking—would be more closely associated with motor coordination (Orefice et al., 2024), whereas other components (e.g., advanced and affective ToM) would be more strongly related to cognitive and linguistic abilities (Baglio et al., 2016; Rosso and Riolfo, 2020; Kaland et al., 2005). Therefore, clarifying the socio-cognitive functioning of children with DCD may provide crucial information for clinical practice. The findings of this study may offer new insights into the development of evidence-based rehabilitation approach to improve the quality of life for this population.

## 2 Methods

### 2.1 Participants

Twenty (20) children with a diagnosis of DCD participated in the study. Participants were aged 7–11 years (M = 8.85, SD = 1.46) and included 8 females and 12 males. All children had received a formal DCD diagnosis according to DSM-5-TR criteria (2022), confirmed by multidisciplinary evaluation (i.e., neuropsychological, psychomotor, and developmental assessment). Children were recruited through local clinical services of the Fondazione Don Carlo Gnocchi in Milan. Inclusion criteria were: (a) a confirmed DCD diagnosis, (b) age between 7 and 11 years, and (c) adequate vision and hearing for task completion. Exclusion criteria were: (a) presence of neurological or genetic conditions, (b) intellectual disability, (c) Autism Spectrum Disorder, (d) Attention Deficit Hyperactivity Disorder, and (e) sensory impairments that could interfere with task performance. Some participants presented with comorbid conditions frequently associated with DCD, including dysgraphia (*n* = 3), Specific Learning Disorders (*n* = 3), and Developmental Language Disorder (*n* = 3), with or without associated literacy difficulties. All participants were native Italian speakers and attended schools. Only children whose parents/legal guardians signed informed consent were included in this study.

Participants were selected according to the convenience sampling method. Ethical approval was waived by the local Ethics Committees because all the procedures being performed were part of the routine care. The study was carried out in accordance with the principles of the Declaration of Helsinki.

### 2.2 Data analysis

All analyses were conducted using JASP (Version 0.19.3; JASP Team, 2025). The analyses were conducted on the specific indices and task scores described in the Measures section. Data were screened for missing values and outliers, with none identified. Given the small sample size and the presence of bounded and ordinal variables, normality assumptions were not met. Therefore, Spearman rank-order correlations were used to examine associations between ToM components and cognitive and motor measures. Partial Spearman correlations were computed controlling for age, gender, and social symptom severity, indexed by the SRS-2 total score dichotomized to identify children with mild-to-severe social difficulties (see Measures section).

To further examine significant associations, multiple linear regression models were estimated for ToM components showing correlational effects. Motor performance scores were entered as primary predictors, with age, gender, and social symptom severity included as covariates. Social symptom severity (SRS-2) was entered as a dichotomous variable based on the established cut-off (>60), distinguishing children within versus below the mild-to-severe range of social symptoms. Given the limited sample size, bias-corrected bootstrap estimates (5,000 resamples) were used for inference, and model fit was evaluated using *R*^2^ and adjusted *R*^2^ values.

### 2.3 Measures

The assessment battery included measures of motor coordination, cognitive abilities, socio-behavioural functioning, and ToM.

#### 2.3.1 Cognitive abilities

Cognitive functioning was assessed using the Italian version of the WISC-IV (Wechsler, 2003; Baron, 2005). The following indices were included in the statistical analyses: Full-Scale IQ (FS-IQ), as a measure of overall cognitive ability, Verbal Comprehension Index (VCI), Perceptual Reasoning Index (PRI), Working Memory Index (WMI), and Processing Speed Index (PSI).

#### 2.3.2 Motor coordination

Motor coordination was assessed using the MABC-2 (Henderson et al., 2007), which provides age-standardized scores. The following MABC-2 scores were included in the analyses: total score, manual dexterity (MD), aiming and catching (AC), and balance (BA).

#### 2.3.3 Socio-behavioural functioning

Socio-behavioural functioning was evaluated using the SRS-2 (Constantino and Gruber, 2012). The SRS-2 yields standardized scores (T-Points) for social awareness (SA), social cognition (SC), social communication (SCom), social motivation (SM), and restricted and repetitive behaviours (RRB), as well as the composite Social Communication and Interaction (SCI) score and a total score reflecting overall social impairment. All these indices were considered in the analyses. Based on the total score and the cut-off criteria (>60), children were classified as falling within or below the mild-to-severe range of social symptoms (*N* = 9), which was used as a control variable in subsequent analyses.

#### 2.3.4 Theory of mind

ToM abilities were assessed using four complementary tasks targeting distinct components of socio-cognitive reasoning. Given the component-based focus of the study, raw scores were analysed in order to preserve variability across these components. To ensure standard administration and mitigate comprehension barriers, the examiner read all written sections of the ToM tests. Specifically, during the Strange Stories Test, the examiner read the narratives while participants tracked the text on their individual copies.

Perspective-taking (PT) was assessed using a task inspired by Dumontheil et al. (2010), in which children were required to infer another person’s visual perspective. Participants were shown a front-facing image of a bookcase containing several objects placed on different shelves, while a person was positioned behind the bookcase and therefore could not see all objects. For each of the five trials, participants were asked to identify the target object (e.g., the smallest or biggest item within a given category, such as candle) from the perspective of the person behind the bookcase. The score was calculated as the total number of correct responses across the five trials (range 0–5), with higher scores indicating better perspective-taking ability.

Understanding of second-order false beliefs (FB-II) was assessed using two standard stories evaluating recursive belief reasoning: The Ice-Cream Van task (Perner and Wimmer, 1985) and the Look-Prediction task (Astington et al., 2002; Liverta Sempio et al., 2001). For each story, children received 1 point for a correct response to the second-order false-belief test question and 1 point for a correct justification of that response, yielding a score ranging from 0 to 2 for each task. Children who failed the three control questions (i.e., memory, realty and first-order false belief) were automatically assigned a score of 0 for that story. A composite second-order false belief score was then calculated by summing performance across the two stories, resulting in a total score ranging from 0 to 4, with higher scores indicating better recursive belief reasoning.

Affective ToM was assessed using a child-adapted version of the Reading the Mind in the Eyes Test (RMET; Baron-Cohen et al., 2001), composed of 28 items. In each trial, children were shown on a computer screen a photograph depicting only the eye region of a face and were asked to select, among four mental-state terms, the one that best described what the person was thinking or feeling. Responses were scored for accuracy, yielding a total score ranging from 0 to 28, with higher scores indicating greater accuracy in recognizing others’ affective states.

Advanced ToM was examined using the Strange Stories (SS) task (Happé, 1994), which assesses children’s ability to interpret non-literal and socially complex scenarios (e.g., sarcasm, persuasion, white lies). Six mental-state stories were administered. Responses were scored on a 3-point scale (2 = full and correct mental-state explanation, 1 = partially correct or incomplete response, 0 = incorrect response). In addition, the presence of mental-state language was coded for each story (1 = present, 0 = absent), resulting in a separate score ranging from 0 to 6. Total scores were computed by summing performance across the six stories, yielding a theoretical range from 0 to 18, with higher scores indicating more accurate mental-state reasoning.

The following ToM scores were included in the analyses: perspective-taking (PT), second-order false belief (FB-II), Reading the Mind in the Eyes Test (RMET), and Strange Stories (SS).

## 3 Results

### 3.1 Descriptive statistics

Descriptive statistics for all cognitive, motor, socio-behavioural, and ToM measures are reported in Table 1. Mean SRS-2 scores were below the clinical cut-off of 60, indicating that, at the group level, socio-behavioural difficulties were generally within the non-clinical range.

**Table 1:** Descriptive statistics for cognitive, motor, socio-behavioural, and ToM measures.

Measure	Possible scores (range)	Mean	SD
Age (months)		113.70	18.68
WISC			
FS-IQ	—	102.90	14.44
VCI	—	108.90	16.02
PRI	—	102.25	18.25
WMI	—	97.00	16.65
PSI	—	92.85	12.93
MABC-2			
Total	1–19	3.90	1.80
MD	1–19	5.25	2.15
AC	1–19	6.45	3.02
BA	1–19	4.40	1.50
ToM			
PT	0–5	4.00	1.37
FB-II	0–4	1.50	1.23
RMET	0–28	16.30	4.19
SS	0–18	9.15	3.87
SRS-2^*^			
SA	37- > 90	55.15	11.09
SC	37- > 90	56.05	12.68
SCom	40- > 90	57.85	12.28
SM	39- > 90	53.35	11.12
RRB	43- > 90	63.10	15.08
SCI	37- > 90	57.4	12.08
Total	38- > 90	59.05	14.56

WISC-IV, Wechsler Intelligence Scale for Children; FS-IQ, Full-Scale IQ; VC, Verbal Comprehension Index; PRI, Perceptual Reasoning Index; WMI, Working Memory Index; PSI, Processing Speed Index; MABC-2, Movement Assessment Battery for Children; MD, manual dexterity; AC, aiming and catching; BA, balance; SRS-2, Social Responsiveness Scale; SA, social awareness; SC, social cognition; SCom, social communication; SM, social motivation; RRB, restricted and repetitive behaviours; SCI, social communication and interaction; ToM, theory of mind; PT, perspective-taking; FB-II, second-order false belief; RMET, reading the mind in the eyes test; SS, strange stories. ^*^For the SRS-2, *T*-scores ≥ 60 indicate clinically relevant mild-to-severe social difficulties.

### 3.2 Correlations

Partial Spearman correlations were computed to examine associations between ToM components and cognitive, motor, and socio-behavioural measures, controlling for age, gender, and SRS-2 severity (mild-to-severe range). Results revealed a component-specific pattern of associations across ToM measures (see Table 2). Perspective-taking (PT) showed a significant positive association with manual dexterity (MD; *ρ* = 0.655, *p* = 0.004), indicating that better fine motor coordination was associated with more accurate perspective-taking, independent of demographic factors and social symptom severity. No significant associations emerged between PT and cognitive indices or other motor domains. Second-order false belief (FB-II) performance was not significantly associated with any cognitive or motor measures. Affective ToM performance (RMET) was positively correlated with verbal comprehension (ICV; *ρ* = 0.509, *p* = 0.037), consistent with the linguistic demands of affective state recognition. Finally, advanced ToM performance assessed through the Strange Stories task (SS) was strongly associated with overall intellectual functioning (FS-IQ; *ρ* = 0.612, *p* = 0.009), verbal comprehension (ICV; *ρ* = 0.546, *p* = 0.023), and working memory (IML; *ρ* = 0.609, *p* = 0.009). No significant associations emerged between SS and motor measures.

**Table 2:** Partial Spearman correlations controlling for age (months), gender, and SRS-2 severity.

Variable	1	2	3	4	5	6	7	8	9	10	11	12
1. FS-IQ	—											
2. CVI	0.749***	—										
3. PRI	0.806***	0.458	—									
4. WMI	0.763***	0.472	0.556*	—								
5. PSI	0.347	0.237	0.365	0.127	—							
6. MD	0.316	0.333	0.404	0.043	0.188	—						
7. AC	−0.210	−0.113	−0.193	−0.383	−0.321	0.145	—					
8. BA	−0.128	−0.007	0.180	−0.201	−0.248	0.367	0.292	—				
9. PT	0.314	0.433	0.399	0.274	−0.125	0.655**	0.005	0.279	—			
10. FB-II	0.291	0.118	0.351	0.226	0.393	0.126	−0.087	−0.116	0.276	—		
11. RMET	0.457	0.509*	0.274	0.280	−0.025	0.353	−0.199	−0.024	0.305	−0.261	—	
12. SS	0.612**	0.546*	0.324	0.609**	0.058	0.095	−0.113	−0.347	0.306	0.140	0.079	—

Bold values indicate statistically significant correlations: *p < 0.05, **p < 0.01; ***p < 0.001.

FS-IQ, Full-Scale IQ; VCI, Verbal Comprehension Index; PRI, Perceptual Reasoning Index; WMI, Working Memory Index; PSI, Processing Speed Index; MD, manual dexterity; AC, aiming and catching, BA, balance; PT, perspective-taking; FB-II, false belief second order; RMET, reading the mind in the eyes test; SS, strange stories.

### 3.3 Regression analysis

To further examine the association between motor skills and perspective-taking, a multiple linear regression was conducted with PT as the dependent variable and MD, age, gender, and SRS-2 severity as predictors. The overall model was significant, *F*(4, 15) = 3.48, *p* = 0.034, explaining approximately 48% of the variance in PT scores (*R^2^* = 0.48; adjusted *R^2^* = 0.34). In the standard regression model, MD emerged as a significant predictor of PT performance. Given the small sample size, robust bias-corrected bootstrap estimates (5,000 resamples) were computed to support inference. In the bootstrap model, MD remained the only significant predictor of PT, whereas age, gender, and SRS-2 severity were no longer significant (see Table 3). This pattern indicates that fine motor coordination uniquely contributes to individual differences in perspective-taking, independently of demographic factors and the severity of social symptoms.

**Table 3:** Multiple linear regression predicting perspective-taking performance.

Predictor	*B*	SE (boot)	*β*	*p*	95% CI
Age	0.04	0.02	0.54	0.079	[−0.01, 0.07]
Gender	−0.29	0.76	−0.13	0.607	[−1.70, 1.28]
SRS-2	1.48	0.80	0.58	0.063	[−0.16, 2.99]
MD	0.44	0.14	0.69	0.023	[0.11, 0.69]

Bootstrap estimates based on 5,000 bias-corrected resamples.

MD, manual dexterity.

## 4 Discussion

The present study examined multiple components of ToM in children with DCD, revealing a component-specific profile rather than a global impairment in ToM abilities. The central finding was a specific association between manual dexterity and perspective-taking. This ToM component was positively related to manual dexterity in partial correlational analyses, controlling for age, gender, and social symptom severity, and this association was confirmed in multiple regression models. Notably, manual dexterity remained the only significant predictor, indicating a selective motor–social link in supporting individual differences in adopting another person’s visual viewpoint.

This finding is consistent with embodied accounts of social cognition, which propose that perspective-taking relies on motor-based simulation and internal visuospatial transformations grounded in bodily experience (Surtees et al., 2013; Reynolds et al., 2015; Tversky and Hard, 2009). Perspective-taking—requiring an understanding that the same scene can be perceived differently from another person’s spatial position—places strong demands on visuospatial transformation processes (Hirai et al., 2020; Gauthier et al., 2018). Children with DCD, who often exhibit impairments in fine motor control and visuospatial processing, may therefore encounter selective difficulties in ToM tasks that depend on these mechanisms (Gauthier et al., 2018; Orefice et al., 2024). Converging evidence supports this interpretation, as reduced performance on visual perspective-taking tasks has been reported in children with DCD (Orefice et al., 2024).

The present findings are also consistent with behavioural evidence indicating that ToM is largely preserved at a general level in DCD, while motor skills—particularly manual dexterity—are more reliably associated with performance-based measures of social cognition (Kilroy et al., 2022). In Kilroy et al. (2022), associations between motor coordination and task-based ToM measures were more evident, whereas dispositional perspective-taking, as assessed by the Interpersonal Reactivity Index, showed limited or inconsistent relationships with motor skills. This pattern suggests that motor–social links in DCD may be more evident when social cognition is assessed through performance-based measures rather than questionnaire-based indices of empathic disposition. Taken together, the present findings, in combination with previous behavioural evidence in DCD (Kilroy et al., 2022; Orefice et al., 2024), are consistent with the possibility that motor constraints selectively impact those aspects of ToM that place greater demands on visuospatial processing, rather than mental-state reasoning more broadly. In line with this interpretation, neuroimaging evidence has shown that socio-cognitive difficulties may arise from selective alterations in embodied simulation processes, even when explicit mental-state reasoning appears relatively intact (Kilroy et al., 2021).

In contrast, other ToM components showed distinct cognitive profiles. Evidence specifically addressing these components in DCD is still limited, and the present findings therefore provide an initial contribution. Advanced ToM was associated with general intellectual functioning, verbal comprehension, and working memory, consistent with patterns reported in typically developing children and reflecting reliance on higher-order linguistic and inferential processes (Baglio et al., 2016; Kaland et al., 2005). Affective ToM was selectively related to verbal comprehension, mirroring findings in typical development and suggesting a primary contribution of semantic processing (Baker et al., 2014; Rosso and Riolfo, 2020). Second-order false belief performance showed no significant associations with cognitive or motor indices, possibly reflecting limited task sensitivity and indicating the need for further investigation with more comprehensive measures. More broadly, the absence of significant motor-related associations across ToM components other than perspective-taking should be interpreted with caution, as the restricted range of motor performance within the clinical DCD sample, together with the relatively small sample size, may have attenuated correlations and reduced statistical power, limiting the detection of weaker or more diffuse associations. Within these constraints, the overall pattern remains compatible with the possibility that advanced, affective, and recursive ToM components rely less directly on motor and visuospatial processes and more on higher-order cognitive and linguistic resources, consistent with evidence of differential motor–social links in DCD (Kilroy et al., 2021).

### 4.1 Implications and conclusions

Overall, the present findings support a differentiated view of ToM in DCD, in which distinct components may rely on partially independent mechanisms. Perspective-taking appears particularly linked to motor coordination and visuospatial transformation processes, whereas advanced and affective ToM are more strongly supported by cognitive–linguistic abilities and appear relatively preserved. This framework may help reconcile previous reports of largely intact ToM on broad measures with the social difficulties often observed in everyday contexts (Kilroy et al., 2021, 2022). From a clinical perspective, these results highlight the importance of multidimensional assessment of social cognition in DCD. Interventions targeting fine motor and visuospatial skills—especially within social and interactive contexts—may support perspective-taking abilities, whereas language- and narrative-based approaches may be more effective for fostering advanced mental-state understanding. Overall, the findings underscore the potential value of targeted, component-specific rehabilitative intervention strategies in DCD.

### 4.2 Limitations and future directions

Several limitations should be acknowledged. First, the relatively small sample size may have limited statistical power and the detection of more subtle associations, underscoring the need for replication in larger and more diverse cohorts. Second, the cross-sectional design precludes conclusions regarding developmental trajectories or causal relationships between motor coordination and ToM, highlighting the importance of longitudinal studies. Third, the absence of a comparison group limits the generalizability of the findings, as it prevents determining whether the observed associations are specific to children with DCD or extend to typically developing populations. Including a control group in future research would also allow a clearer characterization of ToM task performance and a more precise interpretation of ToM abilities in the DCD sample, by making it possible to distinguish disorder-specific features from broader developmental variability. Finally, the restricted range of motor performance within this relatively homogeneous clinical sample, together with the limited sample size, may have attenuated correlations, reducing the likelihood of detecting weaker associations between motor coordination and ToM components other than perspective-taking. Accordingly, these null findings should be interpreted with caution, and future research—including ongoing work within our group—should adopt designs that increase variability in motor and socio-cognitive functioning.

## Data Availability

The raw data supporting the conclusions of this article will be made available by the authors, without undue reservation.

## References

[ref1] American Psychiatric Association (2013). Diagnostic and Statistical Manual of Mental Disorders. 5th Edn American Journal of Psychiatry.

[ref2] American Psychiatric Association (2022). Diagnostic and Statistical Manual of Mental Disorders. Fifth Edition, Text Revision (DSM-5-TR). Washington: American Psychiatric Publishing.

[ref3] Asonitou K. Koutsouki D. Kourtessis T. Kambas A. (2025). Developmental coordination disorder in preschool-aged children: a neuropsychological perspective on visuospatial working memory and attentional, planning, and decision-making processing in relation to fundamental movement skills. Children (Basel). 12:1118. doi: 10.3390/children12091118, 41006983 PMC12468564

[ref4] Astington J. W. Pelletier J. Homer B. (2002). Theory of mind and epistemological development: the relation between children’s second-order false-belief understanding and their ability to reason about evidence. New Ideas Psychol. 20, 131–144. doi: 10.1016/s0732-118x(02)00005-3

[ref5] Baglio G. Blasi V. Sangiuliano Intra F. Castelli I. Massaro D. Baglio F. . (2016). Social competence in children with borderline intellectual functioning: delayed development of theory of mind across all complexity levels. Front. Psychol. 7:1604. doi: 10.3389/fpsyg.2016.01604, 27818637 PMC5073279

[ref6] Baker C. A. Peterson E. Pulos S. Kirkland R. A. (2014). Eyes and IQ: a meta-analysis of the relationship between intelligence and “Reading the mind in the eyes”. Intelligence 44, 78–92. doi: 10.1016/j.intell.2014.03.001

[ref7] Baron I. S. (2005). Test review: Wechsler intelligence scale for children-fourth edition (WISC-IV). Child Neuropsychol. 11, 471–475. doi: 10.1080/09297040590951587, 16306021

[ref8] Baron-Cohen S. Wheelwright S. Hill J. Raste Y. Plumb I. (2001). The “Reading the mind in the eyes” test revised version: a study with normal adults, and adults with Asperger syndrome or high-functioning autism. J. Child Psychol. Psychiatry 42, 241–251. doi: 10.1111/1469-7610.00715 11280420

[ref9] Bianco F. Cornaggia A. Massaro D. Marchetti A. Castelli I. (2025). Desires and beliefs: the development of second-order theory of mind reasoning in preschoolers and in school-age children. Front. Psychol. 16:1525368. doi: 10.3389/fpsyg.2025.1525368, 40115289 PMC11922878

[ref10] Caldwell M. P. Cheung H. Siu T. S. C. (2026). Building blocks through the eyes of others: a training study in visuospatial perspective-taking for improving children's emotion understanding. Child Dev. 97, 83–96. doi: 10.1093/chidev/aacaf007 41757403

[ref12] Constantino J. N. Gruber C. P. (2012). Social Responsiveness Scale, Second Edition (SRS-2). Torrance: Western Psychological Services.

[ref13] De Roubaix A. Roeyers H. van Waelvelde H. Bar-On L. (2024). Social responsiveness in children with developmental coordination disorder. Braz. J. Phys. Ther. 28:100591. doi: 10.1016/j.bjpt.2024.100591, 38394720 PMC10899025

[ref14] Dewey D. Kaplan B. J. Crawford S. G. Wilson B. N. (2002). Developmental coordination disorder: associated problems in attention, learning, and psychosocial adjustment. Hum. Mov. Sci. 21, 905–918. doi: 10.1016/s0167-9457(02)00163-x, 12620725

[ref15] Dumontheil I. Apperly I. A. Blakemore S. J. (2010). Online usage of theory of mind continues to develop in late adolescence. Dev. Sci. 13, 331–338. doi: 10.1111/j.1467-7687.2009.00888.x 20136929

[ref16] Flavell J. (1974). The Development of Inferences about Others T. Mischel Understanding Other Persons Understanding Other Persons. Oxford: Blackwell.

[ref17] Flavell J. H. (1977). The Development of Knowledge about Visual Perception the Nebraska Symposion on Motivation, vol. 25 Lincoln, NE: University of Nebraska Press, 43–76. 753993

[ref18] Flavell J. H. Everett B. A. Croft K. Flavell E. R. (1981). Young children's knowledge about visual perception: further evidence for the level 1–level 2 distinction. Dev. Psychol. 17, 99–103. doi: 10.1037/0012-1649.17.1.99

[ref19] Gauthier S. Anzalone S. M. Cohen D. Zaoui M. Chetouani M. Villa F. . (2018). Behavioral own-body-transformations in children and adolescents with typical development, autism spectrum disorder, and developmental coordination disorder. Front. Psychol. 9:676. doi: 10.3389/fpsyg.2018.00676 29887813 PMC5981221

[ref20] Happé F. G. (1994). An advanced test of theory of mind: understanding of story characters' thoughts and feelings by able autistic, mentally handicapped, and normal children and adults. J. Autism Dev. Disord. 24, 129–154. doi: 10.1007/BF02172093, 8040158

[ref21] Henderson S. E. Sugden D. A. Barnett A. L. (2007). Movement Assessment Battery for Children. 2nd Edn. San Antonio: The Psychological Corporation.

[ref22] Hirai M. Muramatsu Y. Nakamura M. (2020). Role of the embodied cognition process in perspective-taking ability during childhood. Child Dev. 91, 214–235. doi: 10.1111/cdev.13172, 30408152

[ref2001] JASP Team (2025). JASP (Version 0.19.3)[Computer software].

[ref23] Jayashankar A. Bynum B. Butera C. Kilroy E. Harrison L. Aziz-Zadeh L. (2023). Connectivity differences between inferior frontal gyrus and mentalizing network in autism as compared to developmental coordination disorder and non-autistic youth. Cortex 167, 115–131. doi: 10.1016/j.cortex.2023.06.014, 37549452 PMC10543516

[ref24] Kaland N. Møller-Nielsen A. Smith L. Mortensen E. L. Callesen K. Gottlieb D. (2005). The strange stories test: a replication study of children and adolescents with Asperger syndrome. Eur. Child Adolesc. Psychiatry 14, 73–82. doi: 10.1007/s00787-005-0434-2, 15793686

[ref25] Kenny L. Hill E. Hamilton A. F. D. C. (2016). The relationship between social and motor cognition in primary school age-children. Front. Psychol. 7:228. doi: 10.3389/fpsyg.2016.00228 26941685 PMC4764733

[ref26] Kilroy E. Harrison L. Butera C. Jayashankar A. Cermak S. Kaplan J. . (2021). Unique deficit in embodied simulation in autism: an fMRI study comparing autism and developmental coordination disorder. Hum. Brain Mapp. 42, 1532–1546. doi: 10.1002/hbm.25312, 33320398 PMC7927289

[ref27] Kilroy E. Ring P. Hossain A. Nalbach A. Butera C. Harrison L. . (2022). Motor performance, praxis, and social skills in autism spectrum disorder and developmental coordination disorder. Autism Res. 15, 1649–1664. doi: 10.1002/aur.2774, 35785418 PMC9543450

[ref28] Lingam R. Golding J. Jongmans M. J. Hunt L. P. Ellis M. Emond A. (2010). The association between developmental coordination disorder and other developmental traits. Pediatrics 126, e1109–e1118. doi: 10.1542/peds.2009-2789, 20956425

[ref29] Liverta Sempio O. Marchetti A. Lecciso F. (2001). Il SAT Famiglia e il SAT Scuola: Strumenti di Misura dell’Ansia da Separazione da Genitori ed Insegnanti. Milan: Università Cattolica del Sacro Cuore, ISU.

[ref30] Mancini V. O. Licari M. K. Alvares G. A. McQueen M. C. McIntyre S. Reynolds J. E. . (2024). Psychosocial wellbeing, parental concerns, and familial impact of children with developmental coordination disorder. Res. Dev. Disabil. 145:104659. doi: 10.1016/j.ridd.2023.104659, 38160588

[ref31] Miller H. L. Licari M. K. Bhat A. Aziz-Zadeh L. S. van Damme T. Fears N. E. . (2024). Motor problems in autism: co-occurrence or feature? Dev. Med. Child Neurol. 66, 16–22. doi: 10.1111/dmcn.15674, 37332143 PMC10725993

[ref32] Obeid R. DeNigris D. Brooks P. J. (2022). Linking fine motor skills with theory of mind in school-age children. Int. J. Behav. Dev. 46, 542–554. doi: 10.1177/01650254221116863

[ref33] Omer S. Leonard H. C. (2021). Internalising symptoms in developmental coordination disorder: the indirect effect of everyday executive function. Res. Dev. Disabil. 109:103831. doi: 10.1016/j.ridd.2020.103831, 33360963

[ref34] Orefice C. Cardillo R. Lonciari I. Zoccante L. Mammarella I. C. (2024). “Picture this from there”: spatial perspective-taking in developmental visuospatial disorder and developmental coordination disorder. Front. Psychol. 15:1349851. doi: 10.3389/fpsyg.2024.1349851, 38708023 PMC11066165

[ref35] Perner J. Wimmer H. (1985). “John thinks that Mary thinks that…” Attribution of second-order beliefs by 5-to 10-year-old children. J. Exp. Child Psychol. 39, 437–471. doi: 10.1016/0022-0965(85)90051-7

[ref36] Pinero-Pinto E. Romero-Galisteo R. P. Sánchez-González M. C. Escobio-Prieto I. Luque-Moreno C. Palomo-Carrión R. (2022). Motor skills and visual deficits in developmental coordination disorder: a narrative review. J. Clin. Med. 11:7447. doi: 10.3390/jcm11247447, 36556062 PMC9784736

[ref37] Reynolds J. E. Licari M. K. Elliott C. Lay B. S. Williams J. (2015). Motor imagery ability and internal representation of movement in children with probable developmental coordination disorder. Hum. Mov. Sci. 44, 287–298. doi: 10.1016/j.humov.2015.09.012 26457342

[ref38] Rosso A. M. Riolfo A. (2020). A further look at Reading the mind in the eyes-child version: association with fluid intelligence, receptive language, and intergenerational transmission in typically developing school-aged children. Front. Psychol. 11:586065. doi: 10.3389/fpsyg.2020.586065, 33365000 PMC7750633

[ref40] Sartori R. F. Valentini N. C. Fonseca R. P. (2020). Executive function in children with and without developmental coordination disorder: a comparative study. Child Care Health Dev. 46, 294–302. doi: 10.1111/cch.12734, 31845379

[ref41] Schoemaker M. M. Lust J. M. Steenbergen B. Houwen S. Diepstraten J. E. M. Wilson P. H. . (2024). Developmental coordination disorder subtypes also vary in the pattern of behavioral and emotional problems. Front. Psychol. 15:1418295. doi: 10.3389/fpsyg.2024.1418295, 39679145 PMC11638586

[ref43] Smyth M. M. Anderson H. I. (2000). Coping with clumsiness in the school playground: social and physical play in children with coordination impairments. Br. J. Dev. Psychol. 18, 389–413. doi: 10.1348/026151000165760

[ref44] Sumner E. Leonard H. C. Hill E. L. (2016). Overlapping phenotypes in autism Spectrum disorder and developmental coordination disorder: a cross-syndrome comparison of motor and social skills. J. Autism Dev. Disord. 46, 2609–2620. doi: 10.1007/s10803-016-2794-5, 27126816 PMC4938850

[ref45] Surtees A. Apperly I. Samson D. (2013). Similarities and differences in visual and spatial perspective-taking processes. Cognition 129, 426–438. doi: 10.1016/j.cognition.2013.06.008, 23999408

[ref46] Surtees A. D. Butterfill S. A. Apperly I. A. (2012). Direct and indirect measures of level-2 perspective-taking in children and adults. Br. J. Dev. Psychol. 30, 75–86. doi: 10.1111/j.2044-835X.2011.02063.x, 22429034

[ref47] Tversky B. Hard B. M. (2009). Embodied and disembodied cognition: spatial perspective-taking. Cognition 110, 124–129. doi: 10.1016/j.cognition.2008.10.008, 19056081

[ref48] Valle A. Massaro D. Castelli I. Marchetti A. (2015). Theory of mind development in adolescence and early adulthood: the growing complexity of recursive thinking ability. Eur. J. Psychol. 11, 112–124. doi: 10.5964/ejop.v11i1.829, 27247645 PMC4873097

[ref49] van Dyck D. Baijot S. Aeby A. De Tiège X. Deconinck N. (2022). Cognitive, perceptual, and motor profiles of school-aged children with developmental coordination disorder. Front. Psychol. 13:13. doi: 10.3389/fpsyg.2022.860766, 35992485 PMC9381813

[ref50] Wechsler D. (2003). WISC-IV. Wechsler Intelligence Scale for Children – Fourth Edition Technical and Interpretive Manual. San Antonio, TX: The Psychological Association.

[ref51] Wellman H. M. Cross D. (2001). Theory of mind and conceptual change. Child Dev. 72, 702–707. doi: 10.1111/1467-8624.00309, 11405576

[ref53] Yu C. L. Wellman H. M. (2024). A meta-analysis of sequences in theory-of-mind understandings: theory of mind scale findings across different cultural contexts. Dev. Rev. 74:101162. doi: 10.1016/j.dr.2024.101162

